# Supplementary material for: Polyketide synthase gene clusters in the ericoid mycorrhizal fungus Oidiodendron maius: genome-wide prediction, expression profiling, and functional characterization of knockout mutants
Source: Front Plant Sci. 2026 Sep 16;17:1924430. doi: 10.3389/fpls.2026.1924430 (PMC13623903; doi:10.3389/fpls.2026.1924430)
Supplement: Supplementary file 1 [file SupplementaryFile1.zip › Supplementary Materials/Supplementary Folder 1.zip!/Oidma1_AssemblyScaffolds_Repeatmasked/knownclusterblast/cluster52/jgi.p_Oidma1_46342_mibig_hits.html]

| MIBiG Protein | Description | MIBiG Cluster | MiBiG Product | % ID | % Coverage | BLAST Score | E-value |
| --- | --- | --- | --- | --- | --- | --- | --- |
| CCH26291.1 | putative\_O-methyltransferase | BGC0001158\_c2 | Bikaverin\_biosynthetic\_gene\_cluster | 33 | 101.160092807 | 232 | 7.5e-61 |
| ABC79591.2 | cercosporin\_toxin\_biosynthesis\_protein | BGC0001247\_c2 | Cercosporin\_biosynthetic\_gene\_cluster | 32 | 91.1832946636 | 193 | 5.1e-49 |
| BAH23997.1 | O-methyltransferase | BGC0000356 | Fumitremorgin\_biosynthetic\_gene\_cluster | 31 | 83.2946635731 | 143 | 4.6e-34 |
| ANY57889.1 | PenK | BGC0001372 | Penigequinolone\_biosynthetic\_gene\_cluster | 25 | 99.3039443155 | 138 | 1.9e-32 |
| AAS90065.1 | OmtA | BGC0000009 | Aflatoxin\_biosynthetic\_gene\_cluster | 27 | 83.0626450116 | 136 | 5.6e-32 |
| BAE71329.1 | o-methyltransferase\_A | BGC0000004 | Aflatoxin\_biosynthetic\_gene\_cluster | 28 | 83.9907192575 | 136 | 9.6e-32 |
| AAS90041.1 | OmtA | BGC0000008 | Aflatoxin\_biosynthetic\_gene\_cluster | 28 | 83.9907192575 | 135 | 1.3e-31 |
| AAS90087.1 | OmtA | BGC0000010 | Aflatoxin\_biosynthetic\_gene\_cluster | 28 | 83.9907192575 | 134 | 2.1e-31 |
| ACH72896.1 | AflO | BGC0000011 | Aflatoxin/sterigmatocystin\_biosynthetic\_gene\_cluster | 32 | 43.1554524362 | 90 | 4.6e-18 |
| AAD32742.2 | MmcR | BGC0000915 | Mitomycin\_biosynthetic\_gene\_cluster | 28 | 50.3480278422 | 78 | 2.4e-14 |
| AEE65480.1 | putative\_O-methyltransferase | BGC0000223 | Fluostatin\_biosynthetic\_gene\_cluster | 32 | 35.7308584687 | 76 | 6.9e-14 |
| ADI24956.1 | GsfD | BGC0000070 | Griseofulvin\_biosynthetic\_gene\_cluster | 32 | 37.5870069606 | 74 | 3.4e-13 |
